# A Novel Breeding Target for Salt-Tolerant Maize: *ZmEXPA3* Overexpression Enhances Growth of Maize Under Both Non-Stressed and Salt Stress Conditions Through Cell-Wall Architecture Alteration

**DOI:** 10.3390/plants14233697

**Published:** 2025-12-04

**Authors:** Bingying Leng, Xia Liu, Yue Sun, Huiru Yin, Chunhua Mu, Shijun Ma, Qiantong Liu, Jing Hou, Zhenwei Yan, Guoqi Yao

**Affiliations:** 1 Maize Research Institute, Shandong Academy of Agricultural Sciences, Jinan 250100, China; lengbingying@saas.ac.cn (B.L.);; 2 College of Agronomy, Qingdao Agricultural University, Qingdao 266109, China; 3 School of Agriculture, Ludong University, Yantai 264001, China

**Keywords:** cell expansin, maize, ROS clearance, salt tolerance, *ZmEXPA3*

## Abstract

Expansins contribute to maize tolerance to salt stress, but the molecular mechanisms by which they function under high-salinity conditions remain poorly understood. In this research, the α-expansin gene *ZmEXPA3* was characterized. We obtained overexpression transgenic lines in maize and determined physiological and biochemical indices to elucidate its molecular role in salt stress. Our results confirmed that *ZmEXPA3* functioned as a positive salt tolerance regulator and was potentially regulated by abscisic acid (ABA) and methyl jasmonate (MeJA). ZmEXPA3 located to the cytoplasm and cell wall. Overexpression of *ZmEXPA3* achieved thicker cell wall and bigger cell size and thereby promoted biomass accumulation. The *ZmEXPA3-OE* lines showed a marked reduction in malondialdehyde (MDA) and H_2_O_2_ accumulation compared to the WT under salt stress. Overexpression of *ZmEXPA3* elevated the enzyme activity of peroxidase (POD) and superoxide dismutase (SOD) and proline accumulation and decreased the Na^+^/K^+^ ratio in roots. Transcriptome and Gene Ontology (GO) enrichment analysis of *ZmEXPA3-OE* lines and WT showed that many differentially expressed genes (DEGs) were enriched in cell-wall-related terms, plant hormone response, osmotic stress response, salt stress response, oxidoreductase activity, etc. Changes in these processes may be the primary reasons why *ZmEXPA3* overexpression promotes growth and salt tolerance.

## 1. Introduction

The increasing soil salinization poses an enormous threat to food security [1]. Salt stress limits plant productivity and yield in agricultural areas through a cascade of interconnected physiological disruptions, primarily driven by ionic toxicity, osmotic stress, and oxidative damage [2]. It is urgently necessary to improve salinized soil, enhance crop salt tolerance, and increase salinized soil utilization rate. The root system is the first structure of seedlings exposed to salt stress. During this process, the cell wall is the first line of defense to be breached. Salt stress significantly inhibits root growth, leading to shorter roots, reduced surface area, and fewer root tips [3]. The selective loosening of the cell wall brings about wall stress relaxation, thereby enabling the cell to absorb water and expand [4].

Plant growth is tightly constrained by the properties of the cell wall. Expansins are crucial for cell-wall remodeling, including cell-wall expansion and elongation, and are considered the most important cell-wall modifiers for cell extension [5,6,7]. Expansins are involved in many biological processes involving seed germination [8], stomata opening and closing [9,10], fruit softening [11,12], xylem formation [13,14], root hair and lateral root formation [15,16], and biotic [17,18] and abiotic stress responses [19,20,21,22]. The expansins of plants belong to four subfamilies: α-expansin (EXPA), β-expansin (EXPB), expansin-like A (EXLA), and expansin-like B (EXLB) [23,24], and there is another subfamily called expansin-like X (EXLX) in bacteria and fungi [25]. Expansins have two typical domains. Domain 1 at the N-terminal has a six-stranded double-psi beta-barrel (DPBB) similar to glycosyl hydrolase family-45 (GH45) proteins, with a conserved histidine-phenylalanine-aspartate (HFD) motif. Domain 2 at the C-terminal is said to take part in binding cell-wall polysaccharides [26,27].

Increasing studies indicate that expansins play an important part in salt tolerance [28,29]. Heterologous expression of wheat *TaEXPA2* in tobacco conferred enhanced salt tolerance, manifested through promoted primary root elongation, increased lateral root density, and improved leaf chlorophyll retention under salt stress [30]. Heterologous expression of rose *RhEXPA4* altered stomata development of *Arabidopsis thaliana*, resulting in reduced stomatal density and consequently decreased salt sensitivity [31]. *Osmanthus fragrans* expansin-like A gene *OfEXLA1* was markedly induced by salt, and *OfEXLA1* overexpression promoted plant growth and improved the salt tolerance of *Arabidopsis* [22]. *Salix matsudana* Koidz expansin *SmEXPA13* could enhance salt tolerance and was regulated by *SmMYB1R1-L* [32].

Reactive oxygen species (ROS) are a primary agent of damage in plants under salt stress, which manifests primarily as oxidative stress. Enhancing the ROS scavenging capacity is an effective strategy for plants to mitigate salt stress-induced damage [33]. Peroxidases are a group of ubiquitous isozymes involved in plant growth, development, and defense mechanisms [34]. Based on the presence of the heme prosthetic group, the peroxidase superfamily is categorized into heme-containing and non-heme types. Heme-containing peroxidases are further subcategorized into two subgroups: animal and non-animal types. The non-animal heme PRXs are classified into three distinct classes: class I, such as ascorbate peroxidase (APX), cytochrome c oxidase (CcO), and bacterial catalase peroxidase (CAT); class II, such as lignin peroxidase and Mn^+2^ dependent peroxidase; class III, such as horseradish peroxidase (HRP) and glutathione peroxidase (GPX) [35]. Class III peroxidases are pivotal in the biosynthesis and metabolism of several compounds such as the oxidation of lignin, H_2_O_2_ removal, cell elongation and so on [36]. As we know, class III peroxidases have several short names, e.g., PRX, POD, POX, and PER [37]. In maize, PRX is typically used to denote Class III peroxidases and 119 full-length PRX genes were recognized in the B73 maize genome [38]. As a prominent protein in the cell wall, PRXs mediate critical processes such as cell-wall remodeling and the response to environmental stresses. Suppressing *AtPrx72* down-regulated the lignin biosynthesis pathway. In addition, genes and transcription factors crucial for secondary wall thickening were inhibited [39]. Wheat *TaPRX-2A* played a positive role in salt tolerance by increasing ROS clearance [40].

Our previous study also showed that *ZmEXPA6* increased the salt tolerance of *Arabidopsis*. Heterologous overexpression of *ZmEXPA6* raised the accumulation of osmoregulatory substances and antioxidant capacity [41]. Although a lot of studies have shown that expansins take part in stress responses, few studies on the molecular mechanisms of expansins response to salt were carried out in maize.

This research aims to elucidate the molecular mechanisms by which *ZmEXPA3* enhances salt tolerance. Herein, we obtained *ZmEXPA3* overexpression lines and estimated the phenotypic responses, including changes in the biomass and root cellular structure. Overexpression of *ZmEXPA3* increased root cell-wall thickness, cell size, and plant biomass, and influenced the expression of peroxidases. *ZmEXPA3*-OE lines showed decreased ROS accumulation and Na^+^/K^+^ ratio, and increased the enzyme activity of POD and SOD and proline content. These findings elucidated the molecular mechanisms of *ZmEXPA3*, boosting plant growth and improving salt tolerance. In future studies, we can employ transgenic approaches to enhance maize growth and salt tolerance, thereby enabling its cultivation in high-salinity regions for agricultural production.

## 2. Results

### 2.1. Bioinformatics Analysis and Expression Pattern of ZmEXPA3

*ZmEXPA3* consists of a complete 729 bp open reading frame, encoding 263 amino acids. ExPASY analysis determined its molecular mass and isoelectric point to be 27.77 kDa and 8.62, respectively. The secondary structure composition of ZmEXPA3 includes α-helices, extended β-strands, and random coils, with the random coil content representing the largest proportion. ZmEXPA3 revealed a DPBB1 domain located at residues 70–158 and a conserved His-Phe-Asp motif. Transmembrane region prediction showed that ZmEXPA3 is outside the membrane with no transmembrane region. Hydrophobicity prediction showed that ZmEXPA3 is hydrophilic (Figure S1).

**Figure 1:**
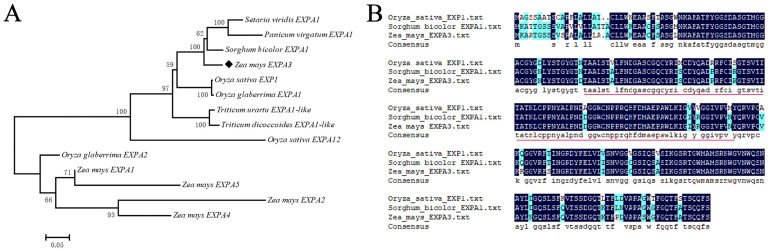
The phylogenetic and structural features of *ZmEXPA3*. (**A**) The phylogenetic tree was constructed based on the amino acid sequence of ZmEXPA3 and its homologs in maize, rice, wheat, etc., using ClustalX1.81 and MEGA7.0 software. (**B**) Alignment of ZmEXPA3 with *Sorghum bicolor* EXPAl and *Oryza sativa* EXP1. The conserved DPBB domains are marked with red lines.

A phylogenetic tree was built on the basis of the amino acid sequence of ZmEXPA3 and its homology in maize, sorghum, wheat, rice, sorghum, *Setaria viridis*, and *Panicum virgatum* (Figure 1A). The comparison between ZmEXPA3, *Sorghum bicolor* EXPA1, and *Oryza sativa* EXP1 showed that the conserved DPBB1 regions have high homology (Figure 1B).

**Figure 2:**
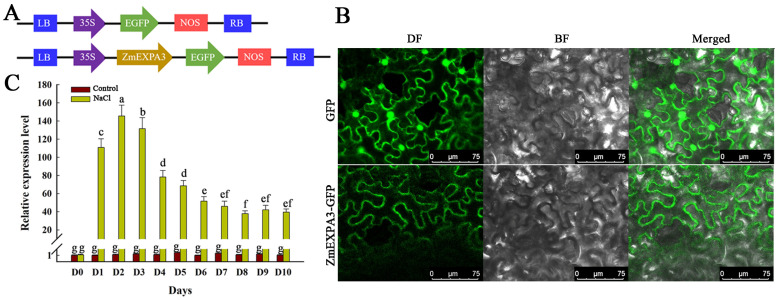
ZmEXPA3 localization and *ZmEXPA3* expression studies. (**A**) Constructs for ZmEXPA3 subcellular localization. (**B**) ZmEXPA3 was localized into cytoplasm and the cell wall. Bar = 75 μm. (**C**) *ZmEXPA3* expression level in WT plants exposed to control and 100 mM NaCl over a 10-day period. Bars (mean ± SD) that do not share a common letter differ significantly (*p* < 0.05) following Duncan’s multiple range test.

In addition, we utilized PlantCARE to analyze the promoter region of the *ZmEXPA3* (2000 bp upstream of the start codon). Sequence analysis showed that the promoter region contained many cis-acting regulatory elements: G-box, abscisic acid responsive element (ABRE), TGACG-motif, CGTCA-motif, which is responsive to light, abscisic acid (ABA) and methyl jasmonate acid (MeJA), etc. (Table S1). Studies reported that some salt-responsive transcription factors like wheat *TabZImethyP15* and tomato *SlAREB1* can recognize ABRE cis-elements and interact with the promoter regions of corresponding genes thereby improving salinity tolerance [42,43]. To detect whether *ZmEXPA3* is responsive to salinity stress, root *ZmEXPA3* expression level in seedlings treated with NaCl over different days was measured. The result showed that *ZmEXPA3* was significantly induced by salinity. The expression level of *ZmEXPA3* increased sharply after salt treatment. Furthermore, prolonged salt treatment resulted in sustained high expression levels (Figure 2C). These outcomes indicated that *ZmEXPA3* is salt-induced.

### 2.2. Overexpression of ZmEXPA3 Promoted Root Development by Facilitating Cell Elongation

**Figure 3:**
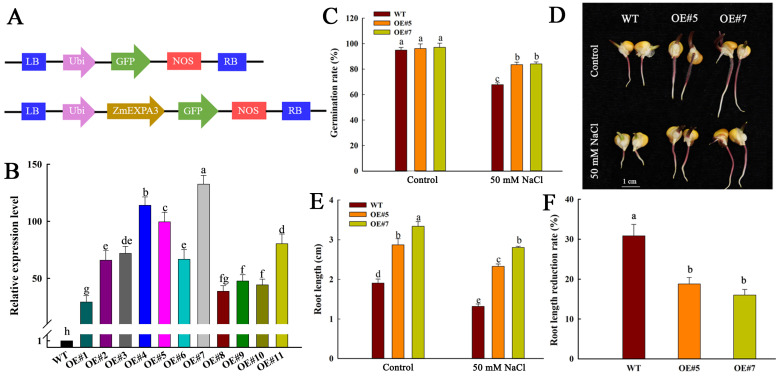
The germination of WT and *ZmEXPA3-OE* under 50 mM NaCl. (**A**) Schematic illustration of constructs used for maize *ZmEXPA3* overexpression line transformation. (**B**) *ZmEXPA3* expression level measured by qPCR. (**C**) Germination rate, analyzed at the seventh day. Data were mean ± SD. (**D**) The root growth phenotype of five-day-old WT, OE#5 and OE#7 seedlings of maize under control and salt treatment. Scale bar = 1 cm. (**E**,**F**) Root length, root length reduction rate. Twenty seedlings were analyzed per line. Value bars (mean ± SD) that do not share a common letter differ significantly (*p* < 0.05) following Duncan’s multiple range test.

To elucidate the biological function of *ZmEXPA3* upon salt stress, we constructed a *ZmEXPA3* overexpression construct driven by a Ubi promoter with a GFP tag (Figure 3A). We selected two high expression lines (OE#5 and OE#7) from the overexpression lines using qPCR at transcript level to carry out subsequent experiments (Figure 3B).

Salt treatment was implemented at the germination stage. Seeds of WT and *ZmEXPA3-OE* were subjected to control and 50 mM NaCl for five days. The seed germination rate between WT and *ZmEXPA3-OE* under control conditions showed no notable difference. NaCl treatment inhibited germination in both WT and OE lines, but the WT exhibited more severe inhibition compared to *ZmEXPA3-OE* (Figure 3C). Under control conditions, *ZmEXPA3-OE* plants exhibited markedly increased root length compared to WT. Salt stress inhibited the root growth of both WT and *ZmEXPA3-OE* plants, resulting in markedly shorter root length relative to control (Figure 3D,E). However, the root length inhibition in *ZmEXPA3-OE* plants was significantly less pronounced than in WT (Figure 3F).

**Figure 4:**
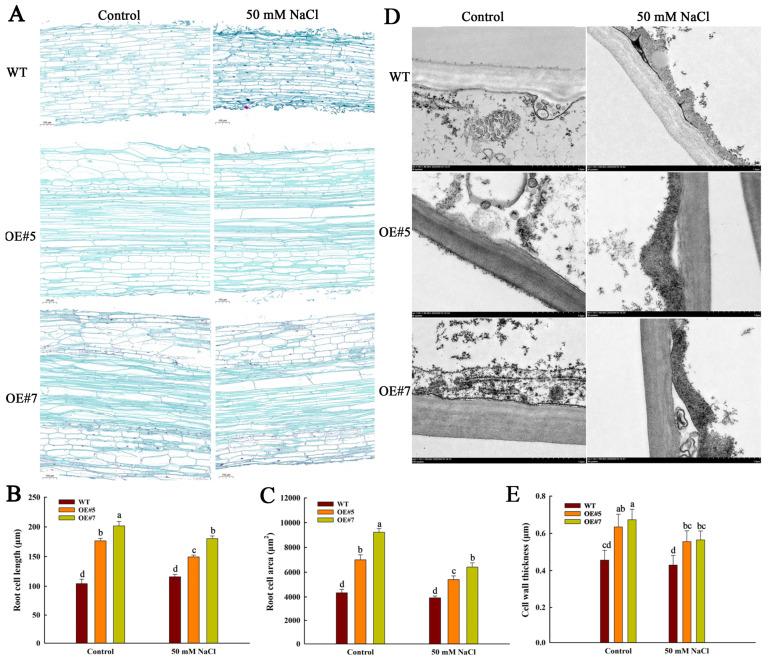
Changes in the tissue organization and cell-wall architecture of the root maturation zone in five-day-old seedlings under control and 50 mM NaCl treatment. (**A**) Longitudinal sections of the root maturation zone captured using a Nikon Eclipse E100. Bar = 100 μm. (**B**,**C**) Root cell length and cell area analyzed with NIS Elements Documentation software (Ver. 4.10). Data are mean ± SD. Diverse letters denote *p* < 0.05 with Duncan’s multiple range test. (**D**) Cell-wall architecture observed through TEM. Bar = 1 μm. (**E**) Cell-wall thickness. Data were mean ± SD. Diverse letters denote *p* < 0.05 with Duncan’s multiple range test.

The development of plant cells determines the morphological changes in plant organs. So, roots’ mature zones during the germination stage were used to explore the mechanisms of *ZmEXPA3* promoting plant growth. We observed longitudinal sections of the roots using paraffin sections, and examined the cell wall using transmission electron microscopy (TEM). The paraffin sections showed that the cell length and area of OE#5 and OE#7 were significantly larger than that of the WT under control conditions. The NaCl treatment group showed a similar trend. After salt stress, the cell length and area of *ZmEXPA3-OE* were obviously lower compared to the control. In contrast, no marked difference was found between WT of NaCl treatment and control (Figure 4A–C). TEM observation revealed that the cell-wall thickness of *ZmEXPA3-OE* was signally larger than that of WT under control conditions. Under NaCl treatment, *ZmEXPA3-OE* still exhibited a thicker cell wall than WT. However, compared with control, cell-wall thickness in the NaCl treatment group was lower (Figure 4D–E). These results demonstrate that *ZmEXPA3* overexpression significantly stimulated cell-wall remodeling, leading to accelerated cell elongation and root development.

### 2.3. Overexpression of ZmEXPA3 Improved Salt Tolerance in Maize Seedling Stages

**Figure 5:**
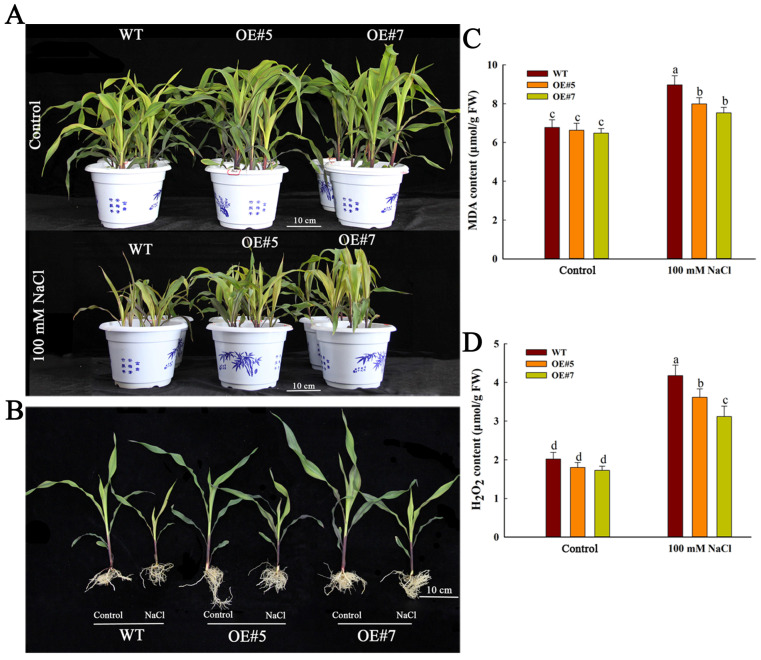
Overexpression of *ZmEXPA3* enhances salt tolerance of seedlings in maize. (**A**,**B**) Growth of WT and *ZmEXPA3-OE* lines under control and 100 mM NaCl treatment. Ten-day-old seedlings were treated with 100 mM NaCl or Hogland’s nutrient solution for twelve days. Scale bar =10 cm. (**C**,**D**) Malondialdehyde (MDA) content, H_2_O_2_ content. Data were mean ± SD. Diverse letters denote *p* < 0.05 with Duncan’s multiple range test.

We extended our investigation to evaluate the salt tolerance of *ZmEXPA3* overexpression lines during the seedling stage. Seedlings of *ZmEXPA3-OE* lines and WT underwent twelve-day exposure to 100 mM NaCl. Evidently, the *ZmEXPA3-OE* plants exhibited significantly distinct growth phenotypes compared to WT plants under control conditions (Hoagland’s nutrient solution). Under control conditions, the *ZmEXPA3-OE* plants outperformed WT in growth metrics (Figure 5A). The *ZmEXPA3-OE* plants developed more robust root systems than WT. Notably, this root system advantage remained significantly greater in *ZmEXPA3-OE* plants than WT even under saline conditions (Figure 5B).

Since MDA content and H_2_O_2_ levels are critical indicators for assessing salt tolerance [44,45], we quantified these indicators in *ZmEXPA3-OE* and WT roots. Under saline conditions, although MDA content in WT and *ZmEXPA3-OE* seedlings was markedly increased compared to that in control, the accumulation of MDA in *ZmEXPA3-OE* seedlings was markedly less than in the WT (Figure 5C). The accumulation of H_2_O_2_ in *ZmEXPA3-OE* lines was slightly lower than that in the WT, with no significant difference observed. Following salt treatment, the H_2_O_2_ content increased significantly in both lines. However, compared to the *ZmEXPA3-OE* lines, the WT accumulated significantly higher levels of H_2_O_2_ (Figure 5D). The overproduced ROS in WT was more serious than those in OE lines. These outcomes demonstrated that overexpression of *ZmEXPA3* reduced the accumulation of ROS.

**Figure 6:**
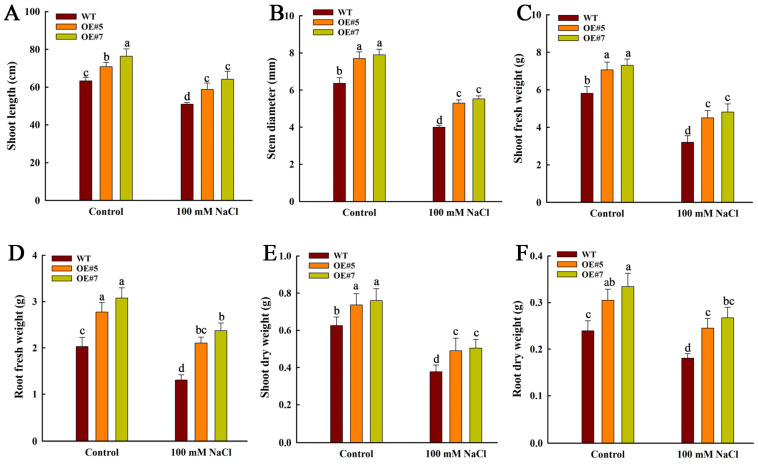
The biomass of WT and *ZmEXPA3-OE* under control and 100 mM NaCL treatment. (**A**) Shoot length. (**B**) Stem diameter. (**C**,**D**) Shoot fresh weight and root fresh weight. (**E**,**F**) Dry weight of shoot and root. Data were mean ± SD. Diverse letters denote *p* < 0.05 with Duncan’s multiple range test.

Consistent with these phenotypic observations, the *ZmEXPA3-OE* plants exhibited significantly greater shoot length and stem diameter compared to WT plants. Although salt stress suppressed both shoot length and stem diameter in all genotypes, the inhibitory effects in WT were markedly more severe than *ZmEXPA3-OE* plants (Figure 6A,B). The overexpression of *ZmEXPA3* significantly enhanced the biomass accumulation of plants. Under control conditions, compared with WT, the fresh and dry weights of shoots and roots in *ZmEXPA3-OE* lines were all notably higher. Under salt stress conditions, these parameters in the *ZmEXPA3-OE* remain significantly higher than WT (Figure 6C–F). Although salt stress inhibited the development of both WT and *ZmEXPA3-OE* plants, the WT showed significantly greater inhibition compared to the *ZmEXPA3-OE* lines (Figure S2). These outcomes demonstrated that overexpression of *ZmEXPA3* could obviously promote the growth of maize plants and improve the salt stress resistance of seedlings.

### 2.4. Overexpression of ZmEXPA3 Improved the Antioxidative Capacity, Osmoregulation and Ion Homeostasis Regulatory Ability

**Figure 7:**
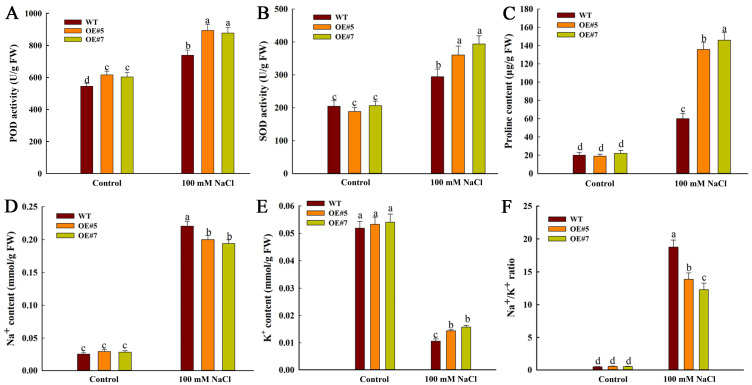
Changes in osmolyte content, enzymatic antioxidant activity, and ion content. (**A**) POD activity. (**B**) SOD activity. (**C**) Proline content. (**D**) Na^+^ content. (**E**) K^+^ content. (**F**) Na ^+^/K^+^ ratio. Data were mean ± SD. Diverse letters denote *p* < 0.05 with Duncan’s multiple range test.

To further investigate the reason for salt tolerance, a series of physiological indicators in roots were measured. Antioxidant enzymes boost salt tolerance via scavenging ROS and thereby reducing oxidative injury. In this study, the POD activity of OE#5 and OE#7 was higher than that of WT under control conditions. Under salt stress, the OE#5 and OE#7 lines also showed elevated POD activity, which was 1.12 and 1.19 times higher than that of the WT, respectively (Figure 7A). In addition, the SOD activity of OE#5 and OE#7 was 1.22 and 1.34-fold higher than that in the WT under salt stress (Figure 7B). The increased activity of POD and SOD demonstrated higher ROS scavenging ability. These results demonstrated that *ZmEXPA3* overexpression boosted antioxidant enzyme activity so as to accelerate ROS clearance.

Salt stress induced a substantial accumulation of proline in both genotypes relative to controls. Notably, the OE#5 and OE#7 exhibited 2.26 and 2.43-fold higher proline content compared to WT under 100 mM NaCl treatment (Figure 7C), suggesting enhanced osmotic adjustment capacity.

To investigate the effects of *ZmEXPA3* on root ion content, the Na^+^ and K^+^ content was determined. No marked difference was detected in Na^+^ content between WT and OE plants under control. Under saline conditions, the OE#5 and OE#7 plants had significantly less Na^+^ excessive accumulation than WT. The Na^+^ content in OE#5 and OE#7 plants showed a 6.82-fold and 6.83-fold increase compared to their control, whereas that in the WT increased 8.74-fold (Figure 7D). Salt treatment resulted in an 72.98% and 70.74% reduction in K^+^ content in OE#5 and OE#7 compared to their control, and a 79.58% reduction in WT compared to its control (Figure 7E). The Na^+^/K^+^ ratio under salt stress in OE#5 and OE#7 was observably lower than WT (Figure 7F). These attested that *ZmEXPA3* overexpression mitigates the detrimental effects of salt stress on plants by maintaining ion homeostasis.

### 2.5. Overexpression of ZmEXPA3 Changed the Expression of Peroxidases Genes

To investigate the molecular mechanisms of *ZmEXPA3* responding to salt in maize at the transcriptional level, the transcriptome profiling of maize *ZmEXPA3-OE#7* (OE) and WT roots under control and 100 mM NaCl was analyzed. A principal component analysis illustrated that samples of NaCl-treated OE were effectively discriminated from samples of control OE, NaCl-treated WT or control WT (Figure S3). These demonstrated that this dataset can be utilized to identify DEGs resulting from *ZmEXPA3* overexpression. There were 2405 DEGs between control OE and WT (OE vs. WT) (|log_2_FoldChange| ≥ 1, *p*-value ≤ 0.01 and *p*-adjust ≤ 0.05), while there were 690 DEGs in NaCl-treated OE compared to WT (NaOE vs. NaWT) (Tables S2 and S3).

**Figure 8:**
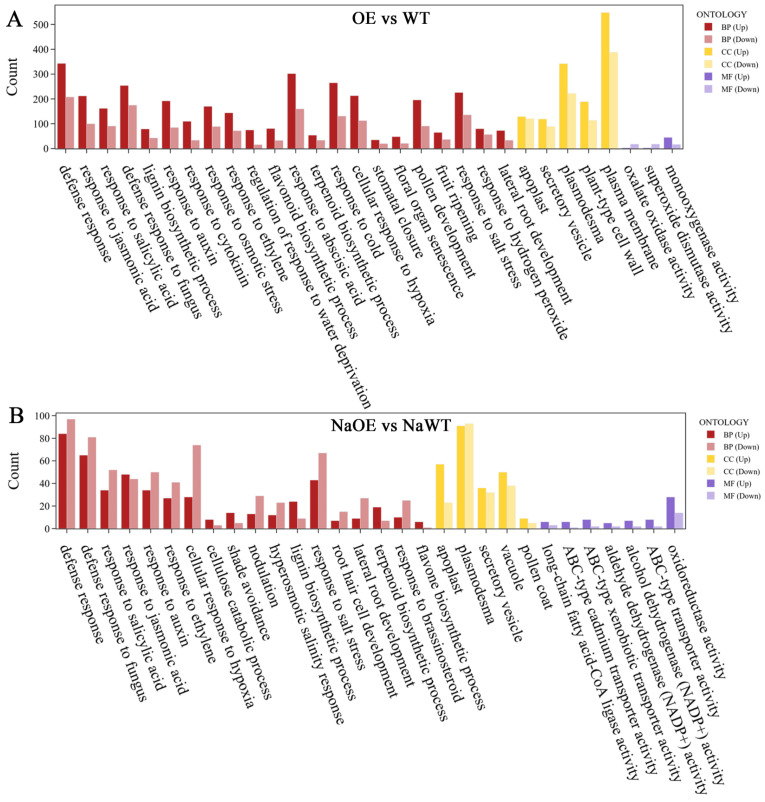
GO enrichment analysis and qPCR validation of DEGs related to *ZmEXPA3*. (**A**) Top over-presented 30 GO terms in the biological process (BP), molecular function (MF), and cellular components (CC) for the 2405 DEGs between control OE and WT (|log_2_FoldChange| ≥ 1, *p*-value ≤ 0.01 and *p*-adjust ≤ 0.05). (**B**) Top over-presented 30 GO terms for the 690 DEGs between 100 mM-treated OE and WT (|log_2_FoldChange| ≥ 1, *p*-value ≤ 0.01 and *p*-adjust ≤ 0.05).

**Figure 9:**
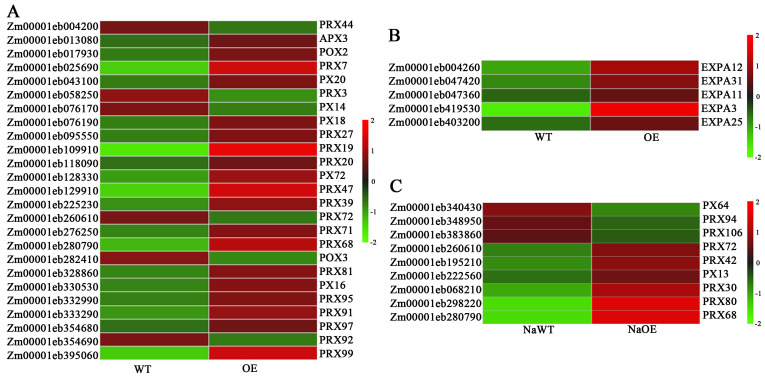
Differentially expressed expansins and peroxidases between OE and WT under control and NaCl treatment. (**A**) Peroxidases under control conditions. (**B**) Expansins under control conditions. (**C**) Peroxidases under NaCl treatment.

GO enrichment analysis of DEGs in OE vs. WT group were determined (Table S4). The 30 enriched GO terms covering the biological process (BP), molecular function (MF), and cellular components (CC) were listed (Figure 8A). GO enrichment analysis of DEGs in NaCl-treated OE and WT were also determined (Table S5) and 30 enriched GO terms were listed (Figure 8B). Many DEGs in the OE vs. WT group were enriched to stress response processes such as response to hydrogen peroxide (GO:0042542, 137 DEGs), superoxide dismutase activity (GO:0042542, 21), response to salt stress (GO:0009651, 100), cellular response to hypoxia (GO:0071456, 152), and so on (Table S4). We conducted an analysis of antioxidant enzyme genes and found that peroxidases were significantly differentially expressed. Twenty-five peroxidases such as *Zm00001eb004200* (*ZmPRX44*, *peroxidase44*), *Zm00001eb013080* (*ZmAPX3*, *ascorbate peroxidase homolog3*), *Zm00001eb017930* (*ZmPOX2*, *guaiacol peroxidase2*), and *Zm00001eb043100* (*ZmPX20*, *peroxidase20*) were found to be significantly differentially expressed, and the vast majority of them were up-regulated (Figure 9A). In addition to *Zm00001eb419530* (*ZmEXPA3*), expansins such as *Zm00001eb004260* (*ZmEXPA12*), *Zm00001eb047420* (*ZmEXPA31*), *Zm00001eb047360* (*ZmEXPA11*) and *Zm00001eb403200* (*ZmEXPA25*) showed significantly higher expression levels in OE plants (Figure 9B).

In the NaOE vs. NaWT group, many DEGs were also enriched to stress response processes such as cellular response to hypoxia (GO:0071456, 102), hyperosmotic salinity response (GO:0042538, 35), reactive oxygen species metabolic process (GO:0072593, 22), response to salt stress (GO:0009651, 110), cellular response to water deprivation (GO:0042631, 33), aldehyde dehydrogenase (NADP+) activity (GO:0033721, 7), oxidoreductase activity (GO:0016717, 9), and so on. There were also many peroxidases such as *Zm00001eb260610* (*ZmPRX72*), *Zm00001eb195210* (*ZmPRX42*), *Zm00001eb280790* (*ZmPRX68*), *Zm00001eb068210* (*ZmPRX30*), *Zm00001eb298220* (*ZmPRX80*), *Zm00001eb222560* (*ZmPX13*), and three genes with reduced expression (Figure 9C). These results demonstrated that overexpression of *ZmEXPA3* influenced the expression of PRXs, thereby enhancing the clearance of ROS.

**Figure 10:**
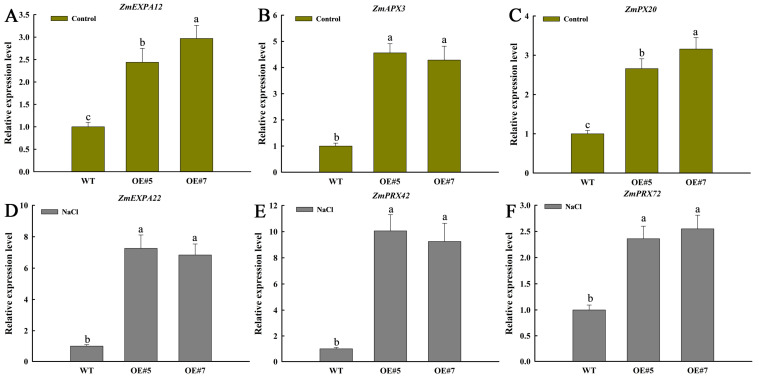
The qPCR verification of DEGs in WT and *ZmEXPA3-OE* plants. (**A**–**C**) Relative expression of *ZmEXPA12*, *ZmAPX3* and *ZmPX20* under control conditions. (**D**–**F**) Relative expression of *ZmEXPA22*, *ZmPRX42*, and *ZmPRX72* under NaCl treatment. Data were mean ± SD. Diverse letters denote *p* < 0.05 with Duncan’s multiple range test.

The qPCR of some DEGs including expansins and peroxidases under control (*ZmEXPA12*, *ZmAPX3*, *ZmPX20*) and salt stress (*ZmEXPA22*, *ZmPRX42*, *ZmPRX72*) conditions were carried out in the WT, OE#5, and OE#7 lines. The results revealed that these genes were significantly up-regulated in *ZmEXPA3-OE* lines, which is consistent with the transcriptomic analysis (Figure 10).

### 2.6. Plant Hormones Potentially Took Part in Regulating ZmEXPA3 Expression Under Salt Stress

Hormones make important contributions to stress response in plants. Plants employ diverse strategies involving hormonal adjustments in both synthesis and signal transduction to cope with salt stress [46]. GO enrichment analysis revealed that compared with WT, a significant number of DEGs in OE were enriched to plant hormone response terms containing jasmonic acid (GO:0009753, 312), salicylic acid (GO:0009751, 253), auxin (GO:0009733, 277), cytokinin (GO:0009735, 144), ethylene (GO:0009723, 216), and abscisic acid (GO:0009737, 462) (Figure 9A). DEGs involved in JA terms like *Zm00001eb314010* (*ZmZIM28*, *ZIM-transcription factor 28*), *Zm00001eb005980* (*ZmZIM26*), *Zm00001eb084980* (*ZmZIM33*) were significantly differentially expressed. DEGs involved in auxin terms like *Zm00001eb301590* (*ZmIAA32*, *Aux/IAA-transcription factor32*), *Zm00001eb239550* (*ZmIAA23*), *Zm00001eb295580* (*ZmIAA47*) and *Zm00001eb326420* (*ZmAAS8, Auxin amido synthetase8*) displayed significantly increased expression. DEGs involved in ABA terms like *Zm00001eb366900* (*ZmbZIP75*), *Zm00001eb147240* (*ZmABRE1*, *ABA-responsive cis-element binding protein1*), *Zm00001eb314900* (*ZmABH4, abscisic acid 8’-hydroxylase4*), *Zm00001eb359740* (*ZmWRKY42*) and *Zm00001eb218880* (*ZmWRKY109*) displayed significantly increased expression.

Under NaCl treatment, compared with WT, many DEGs of OE were also enriched to plant hormone response terms containing salicylic acid (GO:0009751, 86), jasmonic acid (GO:0009753, 92), auxin (GO:0009733, 84), ethylene (GO:0009723, 68), and brassinosteroid (GO:0009741, 35). These results suggested that the enhancement of salt stress tolerance by *ZmEXPA3* overexpression may be mediated by plant hormones.

## 3. Discussion

In this study, we proved that *ZmEXPA3* acted as a positive regulator for the salt tolerance of maize. ZmEXPA3 is a cytoplasmic, plasma membrane, and cell-wall-localized protein. *ZmEXPA3* was strongly induced by salt stress and was mediated by plant hormones such as MeJA and ABA. Overexpression of *ZmEXPA3* promoted root architecture, biomass accumulation, and plant growth by loosening cell walls and stimulating cell expansion, while also maintaining cell elongation capacity under salt stress to enhance tolerance. Overexpression of *ZmEXPA3* changed the expression of peroxidase genes so as to enhance antioxidant activity. In addition, overexpression of *ZmEXPA3* increased osmolyte accumulation and ion homeostasis capacity to improve salt tolerance.

Studies showed that *Osmanthus fragrans* ABI5 (AREB/ABF family)-like genes *OfABL4* and *OfABL5* positively regulate *OfEXLA1* via ABA signaling pathway [22]. The endogenous addition of MeJA significantly promoted the expression of *Eustoma grandiflorum EgEXPA2* and *EgEXPA3* in the petals [47]. Salicylic acid alleviates salt-induced harm in *Capsicum annuum* by regulating biochemical characteristics and certain crucial antioxidant substances [48]. The promoter of *ZmEXPA3* contained multiple cis-acting elements involved in the MeJA and ABA responsiveness (Table S1). And a significant increase in *ZmEXPA3* expression was observed after NaCl treatment (Figure 2C). In addition, GO enrichment analysis of WT and OE under control and salt stress revealed significant enrichment in the DEGs that were involved in plant response terms such as jasmonic acid (GO:0009753), salicylic acid (GO:0009751), auxin (GO:0009733), and abscisic acid (GO:0009737) (Figure 8). These results indicated that *ZmEXPA3* conferred enhanced salt tolerance, and this enhancement may be regulated by hormones, especially the ABA and MeJA signaling pathways.

Root architecture is directly related to the salt stress response of plants, and the cell wall is the very first barrier that directly confronts salt stress [49]. What is more, the cell wall is a crucial determinant of cell size [50]. Expansins are involved in regulating cell-wall loosening, and this helps maintain cell-wall elasticity under osmotic stress and allows cells to expand and maintain turgor at a lower internal water potential, thereby facilitating water uptake under challenging conditions [5,22]. Heterologous expression of *Saccharum SacEXP32* increased the cell area of *Nicotiana benthamiana* [51]. The silencing of *HvEXPA1* suppressed root cell elongation of barley (*Hordeum vulgare*) [16]. Overexpression of the *NtEXPA11* in tobacco not only promoted root system development but also significantly increased leaf area and internode length, consequently improving plant salt tolerance [52]. Our research exhibited that *ZmEXPA3* overexpressing raised the cell-wall thickness and facilitated root cell length and cell area (Figure 4). The overexpression of *ZmEXPA3* altered the root architecture and resulted in a more prosperous root system. In addition, OE lines possessed sturdier shoots (Figure 5B). As a result, OE lines amassed more biomass than WT (Figure 6). GO enrichment analysis in the control OE vs. WT group also revealed significant enrichment in the DEGs that were involved in the plant-type cell wall (GO:0009505), cell-wall organization (GO:0071555), and other cell-wall-related processes (Table S4). These data proved that overexpression of *ZmEXPA3* promoted plant growth by altering the cell-wall structure and loosening to maintain water uptake, thereby enhancing salt stress.

The massive production of ROS is a serious hazard caused by salt stress. Antioxidants are one of the primary methods for scavenging ROS and mitigating the damage caused by stress [53]. The content of MDA reflects the severity of cell damage caused by ROS [54]. Studies showed that *OsEXPA7* overexpression enhanced the salt tolerance of rice by reducing ROS accumulation [55]. Overexpression of wheat *TaEXPA2* decreased the ROS accumulation [56]. In this research, *ZmEXPA3-OE* lines exhibited lower levels of H_2_O_2_ and MDA content (Figure 5C,D), and higher activities of POD and SOD under salt stress (Figure 7C). These outcomes demonstrated that *ZmEXPA3-OE* lines experience less severe oxidative stress and possess a stronger ROS scavenging capacity. Transcriptome analysis revealed that many peroxidases were differentially expressed under control and salt stress conditions in the *ZmEXPA3-OE* line (Figure 9). Studies showed that peroxidases are reported to participate in salt tolerance. Overexpression of *AtPRX3* improved salt tolerance [57]. Soybean *GsPRX9* improved the salt tolerance and antioxidant ability of plants [58]. Heterologous overexpression of Class I peroxidases *SbpAPX* (ascorbate peroxidase) of *Salicornia brachiata* improved the salt tolerance of peanuts [59]. This implies that one mechanism by which *ZmEXPA3* overexpression enhanced ROS clearance was through affecting the peroxidase pathway.

Osmoregulatory substances are essential for plants to cope with salt stress. Proline, as an important osmotic regulatory substance, can regulate osmotic balance through helping to restore plant water content so as to enhance the salt stress resistance of plants [60]. In this paper, *ZmEXPA3-OE* lines of maize possessed obviously higher proline content than WT under salt stress (Figure 7C). Our previous study showed that heterologous *ZmEXPA6* overexpression increased the generation of proline and anthocyanins so as to maintain cellular osmotic homeostasis [41]. In this research, GO enrichment analysis of DEGs in the OE vs. WT group and the NaOE vs. NaWT group also revealed a significant enrichment of the DEGs that were involved in anthocyanin-related processes such as proanthocyanidin biosynthetic process (GO:0010023) and anthocyanin-containing compound biosynthetic process (GO:0009718) (Tables S4 and S5). Anthocyanins also function as a type of osmotic regulator [61]. Not only that, anthocyanins also act as ROS scavengers through their antioxidative properties to resist salt stress [62]. These data confirm that *ZmEXPA3* helps maintain osmotic balance by promoting the accumulation of osmoregulatory substances.

The accumulation of excess sodium ions disrupts ionic homeostasis and causes ion toxicity [63]. Reducing Na^+^ levels and increasing K^+^ content in plants is an effective strategy for enhancing survival probability [64]. Overexpression of wheat *TaEXPB3* enhanced Na^+^ extruding and decreased Na^+^/K^+^ ratio, thus improving salt tolerance of rice [65]. Overexpression of *OsEXPA7* reduced Na^+^ accumulation [55]. In our research, overexpression of *ZmEXPA3* also increased Na^+^/K^+^ ratio of maize. These results indicated that *ZmEXPA3* maintained ionic homeostasis through the modulation of the Na^+^/K^+^ ratio. The overexpression of *ZmEXPA3* contributed to improved ion homeostasis (reduced Na+/K+ ratio), which in turn mitigated secondary oxidative stress. This attenuated stress level was reflected in the modulated responses of the antioxidant system (POD and SOD activity) and the osmotic adjustment system (proline content).

## 4. Materials and Methods

### 4.1. Plant Materials and Growth Conditions

Maize seeds of WT (KN5585) and overexpression lines were used in this experiment. Plants of maize overexpression lines were obtained from Wimi Biotechnology (Changzhou, China). Full and uniform seeds of WT and two overexpression lines (*ZmEXPA3-OE#5* and *ZmEXPA3-OE#7*) were selected to conduct the experiment. Seedlings were cultivated in phytotron (16/8 h, day/night; 600 µmol m^−2^ s^−1^ light intensity, full spectrum; 28 ± 2/22 ± 2 °C, day/night; 65–70% relative humidity).

To explore the effect of salt stress on seed germination, seeds of WT, OE#5, and OE#7 were sterilized by 1% (*v*/*v*) NaClO shaking for 15 min and rinsed with ddH_2_O for 3 times. After that, seeds were transferred to sterilized filter paper in Petri dishes (13 cm square Petri dish, 40 seeds per dish). Distilled water and 50 mM NaCl solution (40 mL per dish) were used for the control and treatment groups. Five-day-old seedlings were photographed and root length was measured. Germination rate was recorded on the seventh day. Each treatment group contained five dishes.

In addition, to inquire into the affects of salt on seedling stages, seeds of WT, OE#5, and OE#7 were planted in vermiculite and covered with cling film for four days. After removing the cling film, seedlings were irrigated with pure water every day. Seven days later, uniform seedlings gently cleaned were transplanted into plastic pots (9 cm × 21 cm × 18 cm, four seedlings per pot) with cleaned sand, and each pot of seedlings was watered with 300 mL of Hogland’s nutrient solution every day. Seedlings were treated with 100 mM NaCl at ten days (Hogland’s nutrient solution as the medium) or Hogland’s nutrient solution every day for twelve days (500 mL per pot). The seedlings were photographed and then cleaned. Each treatment group contained eight pots, and three replicates were performed.

### 4.2. Bioinformatics and Phylogenetic Analysis

The primary physicochemical properties of ZmEXPA3 was analyzed by Protparam (https://web.expasy.org/translate/ accessed on 1 December 2024) and Protparam (https://web.expasy.org/protparam/ accessed on 1 December 2024). SMART (https://smart.embl.de/ accessed on 1 December 2024) was employed for assessing the conserved domains. TMHMM (https://services.healthtech.dtu.dk/services/TMHMM-2.0/ accessed on 1 December 2024) was utilized for determining transmembrane domains. ProtScale (https://web.expasy.org/protscale/ accessed on 1 December 2024) was applied to predict protein hydrophobic properties and hydrophilicity. PlantCARE was employed to forecast the cis-acting elements in promoter. A neighbor-joining phylogenetic tree was built with MEGA 7.0 and ClustalX 1.81, utilizing the software’s default pairwise and multiple alignment parameters. The multiple sequence alignments were carried out by DNAMAN.

### 4.3. Subcellular Localization of ZmEXPA3

Coding sequences of *ZmEXPA3* excluding stop codon were cloned into pCAMBIA1300 vector which has EGFP and driven by 35S promoter. The 35S:ZmEXPA3:EGFP and 35S:EGFP were transferred into tobacco. The EGFP fluorescence signals were observed by DMI8 laser scanning confocal microscope (Leica, Germany). Primers used in subcellular localization experiment were presented in Table S6.

### 4.4. Expression Analysis Under Different Salt Treatment Durations

Seeds of KN5585 were planted in vermiculite for seven days. Gently rinsed seedlings were grown under hydroponic conditions in the Hoagland’s nutrient solution and placed in the 100 mM NaCl solution (using Hogland’s nutrient solution as the medium)) after three days. The roots of seedlings treated with 100 mM NaCl for 0–10 days were washed with distilled water, and the clean roots were dried with absorbent paper and stored in liquid nitrogen. The total RNA and cDNA were prepared according to the protocols of FastPure^®^ Universal Plant Total RNA Isolation and HiScript^®^ III RT SuperMix for qPCR (+gDNA wiper) Kit (Vazyme, Nanjing, China), respectively.

### 4.5. Root Paraffin Sectioning and TEM

Paraffin sectioning was conducted following our established protocol [66]. Briefly, the root maturation zone of maize WT, OE#5, and OE#7 seedlings treated with 50 mM NaCl for seven days were cleaned and put in FAA fixative for 24 h. After dehydration and transparency, embedded samples were sectioned, double-stained (safranin/fast green), and mounted in neutral gum. Sections were finally examined with Nikon Eclipse E100 (Shanghai, China). Cell length and cell area were measured using the measuring tool of the NIS Elements Documentation software. Ten biological replicates were performed.

TEM sample preparation was conducted following the protocol of Lian et al. [67]. The maize root maturation zones of WT, OE#5, and OE#7 seedlings treated with 50 mM NaCl for seven days were cleaned and put in a fixative solution (2.5% glutaraldehyde). Samples were subjected to continuous vacuum extraction to ensure complete submersion. After 2 h fixation at room temperature, the tissues were washed three times using 0.1 mol/L phosphate-buffer solution (PBS, pH 7.4). Post-fixation with 1% osmium tetroxide was performed for 7 h. Samples were then rinsed three times with 0.1M PB, 15 min each. After dehydration, infiltration embedding, polymerization, ultrathin sectioning, and staining, the samples were observed under a TEM (HITACHI, Tokyo, Japan, HT7800). Ten biological replicates were performed.

### 4.6. Measurement of MDA Concentration

Our previous protocol was employed to determine MDA concentration [68]. The roots (0.5 g) of seedlings were used to determine the content of MDA. The absorbance of the supernatant was measured at 532 and 450 nm. Three replicates were performed in this experiment.

### 4.7. Determination of H_2_O_2_

The H_2_O_2_ concentration was determined by titanium sulfate spectrophotometry [69]. Briefly, roots (0.1 g) was homogenized using 0.5 mL PBS in an ice bath. After centrifugation, 250 μL supernatant was mixed with 25 μL titanium sulfate solution (50 mg/mL) and 50 μL concentrated ammonium hydroxide. The precipitate retained after centrifugation was dissolved by 250 μL sulfuric acid (2 mol/L). Absorbance was determined at 415 nm. Three replicates were performed in this experiment.

### 4.8. Measurement of Proline Content

Acidic ninhydrin colorimetric way was applied following Mansour and Ali’s method [70]. Briefly, roots (0.1 g) were combined with 3% sulfosalicylic acid (1 mL). Then, the mixture was extracted through constant shaking at 90 °C for 10 min. The centrifuged supernatant (250 μL) was mixed with 250 μL glacial acetic acid and 2.5% acidic ninhydrin solution and kept in a 92 °C water bath for 30 min. Subsequently, 500 μL of toluene was added, followed by vortexing for 30 s and a brief incubation. The extract absorbance was determined at 520 nm.

### 4.9. Measurement of Antioxidant Enzyme Activities

POD activity was measured by the guaiacol colorimetric method, using 2-methoxyphenol (guaiacol) as the substrate. The absorbance change caused by guaiacol was measured at 470 nm. The activity of SOD was determined by WST-8 (a water-soluble tetrazolium salt) method [71]. WST-8 can react with O_2_^−^ catalyzed by xanthine oxidase to produce formazan dye, with absorbance measured at 450 nm. The SOD and POD extraction were all performed following the manufacturer’s protocol (Mlbio, Shanghai, China).

### 4.10. Determination of Ions

Na^+^ and K^+^ content was determined by flame photometry according to Nada et al. [72]. Concisely, roots (0.1 g dry weight) were digested by nitric acid overnight. Then, samples were oven-dried, and quantitatively transferred into a measuring flask (25 mL). Na^+^ and K^+^ concentration was determined by flame photometer (AP1500, China). Five replicates from five independent roots were used for each stand.

### 4.11. Transcriptome Analysis

Roots of maize WT and OE#7 seedlings treated with 100 mM NaCl or control for twelve days were used to extract total RNA. The experiment was performed with three biological replicates. High-throughput sequencing (Illumina High-Seq2000, Tsingke, Beijing, China) generates raw image data files, which are then converted into raw sequencing data through base-calling analysis using CASAVA. After quality control of the raw data, clean reads were aligned to the reference genome using HISAT2 to obtain their positional information on the genome or genes, as well as sequence-specific features of the sequenced samples [73]. After alignment, the number of reads mapped to each gene (from start to end) was counted based on their positional information in the reference genome. Gene expression levels were quantified using StringTie, with normalization performed via both FPKM and TPM methods [74]. DESeq2 software (v. 1.40.0) were used for differential expression analysis, with a significance threshold set at a |log2(fold change)| > 1, *p*-value ≤ 0.01, and *p*-adjust < 0.05 [75]. GO enrichment analysis were also performed.

### 4.12. qPCR

The qPCR was carried out by employing advanced ChamQ Universal SYBR qPCR Master Mix purchased from Vazyme using a 7500 Real-Time PCR system (Waltham, MA, USA). Three biological replicates were carried out. *ZmTUBULIN* served as an internal control gene [76]. The primers used for qPCRs are listed in Table S6.

### 4.13. Data Analysis

Statistical analysis was performed using SPSS v16.0. Data were analyzed by one-way ANOVA (Duncan’s multiple range test). Significant differences (*p* < 0.05) are denoted by different letters. A minimum of three independent biological replicates were employed for all experiments.

## 5. Conclusions

This study elucidated the molecular mechanisms of *ZmEXPA3* overexpression enhanced plant growth and salt tolerance in maize. Our data indicated that *ZmEXPA3* was induced by salt stress, thereby promoting plant growth and root development. Overexpression of *ZmEXPA3* regulated cell size by altering cell-wall building and enhance salt tolerance of plants by decreasing ROS accumulation and maintaining osmotic equilibrium and ionic homeostasis. The *ZmEXPA3* overexpression potentially changed the peroxidase pathway to facilitate peroxidase-mediated ROS scavenging. The enhancement of salt stress tolerance by *ZmEXPA3* was possibly modulated by hormones such as MeJA and ABA. These results provide compelling evidence that *ZmEXPA3* represents a potential candidate gene for maize improvement aimed at salt tolerance. It is expected to be applied to enhance maize production in saline land and improve utilization efficiency.

## Data Availability

Data will be made available on request.

## Supplementary Materials

The following supporting information can be downloaded at: https://www.mdpi.com/article/10.3390/plants14233697/s1, Figure S1: Bioinformatic analysis of ZmEXPA3; Figure S2: Reduction ratio of shoot length, stem diameter, shoot fresh weight, root fresh weight, shoot dry weight and root dry weight; Figure S3: Principal component analysis. Table S1: Promoter cis-acting element analysis of ZmEXPA3.;Table S2: DEGs of OE compared to WT under control conditions; Table S3: DEGs of OE compared to WT under 100 mM NaCl treatment; Table S4: Top GO terms for DEGs of OE compared to WT under control conditions; Table S5: Top GO terms for DEGs of OE compared to WT under 100 mM NaCl treatment; Table S6: Primers used in this study.
