# Supplementary material for: A Novel Breeding Target for Salt-Tolerant Maize: ZmEXPA3 Overexpression Enhances Growth of Maize Under Both Non-Stressed and Salt Stress Conditions Through Cell-Wall Architecture Alteration
Source: Plants (Basel). 2025 Dec 4;14(23):3697. doi: 10.3390/plants14233697 (PMC12693746; doi:10.3390/plants14233697)
Supplement: Supplementary file 1 [file plants-14-03697-s001.zip › Supplementary Figure S.pdf]

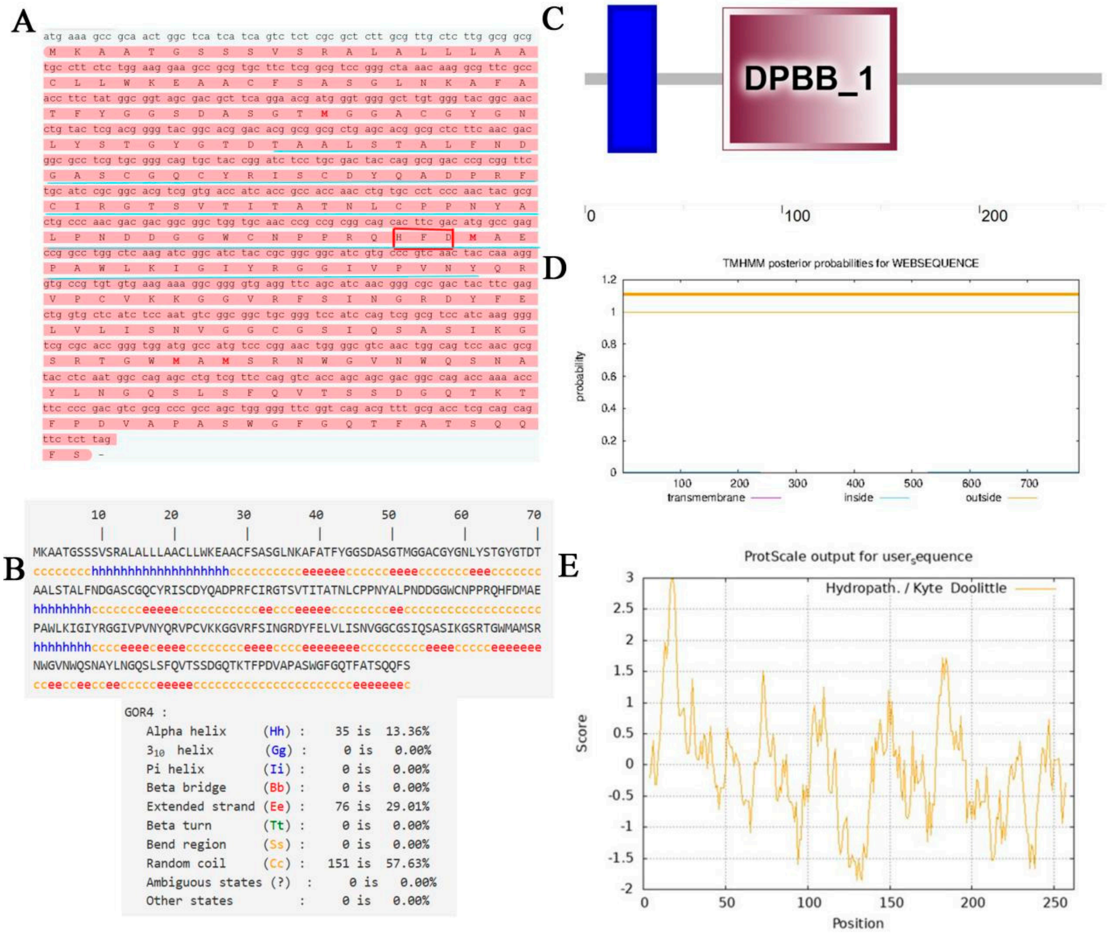

Figure. S1. Bioinformatic analysis of ZmEXPA3. (A) Nucleotide and amino acid residue sequences of ZmEXPA3 analyzed by DNAman. (B) The secondary structure of ZmEXPA3 analyzed by PRABI. (C) Conserved domain of ZmEXPA3 containing a DPBB_1 domain located at amino acids 70-158 drawn with SMART. (D) Prediction of transmembrane conditions analyzed by TMHMM. (E) Hydrophilic prediction analyzed by Expasy ProtScale.

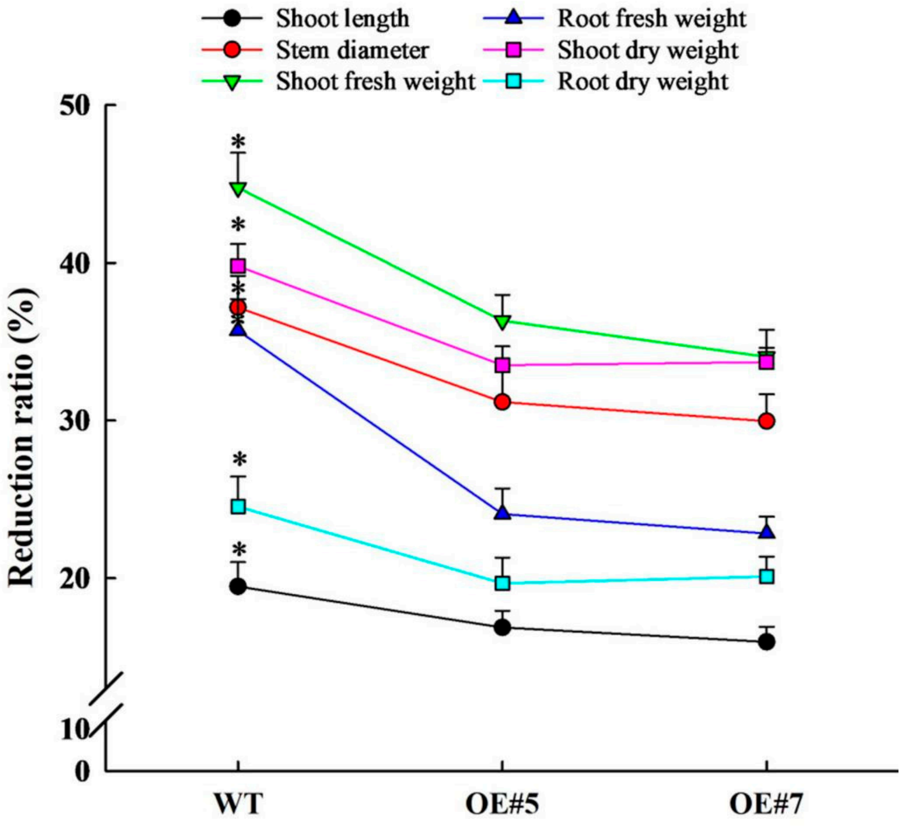

Figure. S2. Reduction ratio of shoot length, stem diameter, shoot fresh weight, root fresh weight, shoot dry weight and root dry weight. Data are means ± SD (n=3). * indicates P<0.05 with Student's t-test.

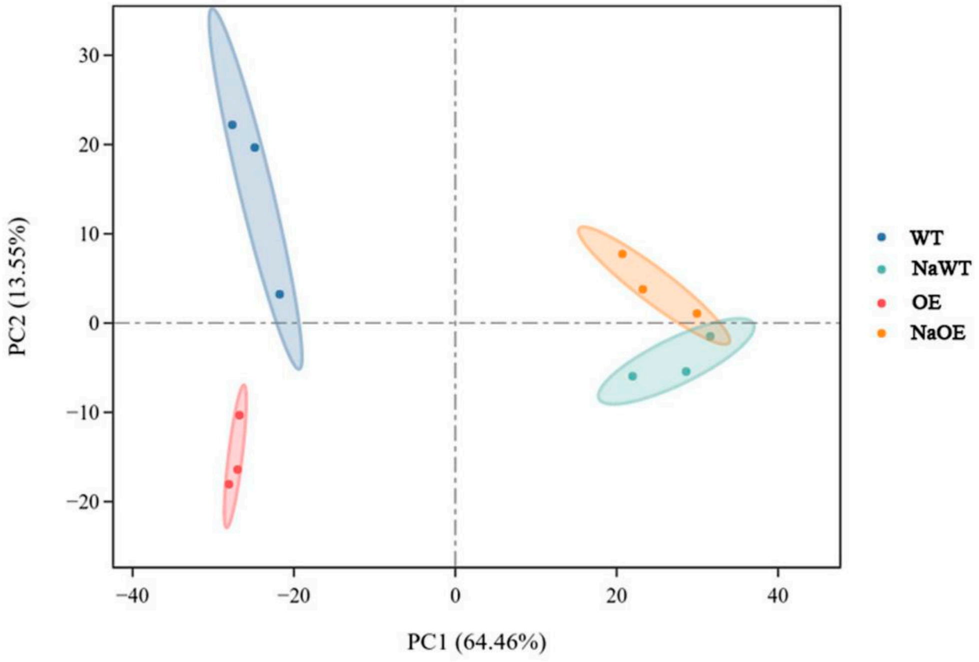

Figure. S3. Principal component analysis. For transcriptome assays, ten-day-old

seedlings of WT and OE7 of maize were irrigated with Hoagland's nutrient solution or 100 mM NaCl for 12 days. Each sample had 20 seedlings. Normalized gene counts with a variance stabilizing transformation in the software DESeq2 were used in the analysis.
